# Design and Implementation of the E-Switch for a Smart Home

**DOI:** 10.3390/s21113811

**Published:** 2021-05-31

**Authors:** Fabian García-Vázquez, Héctor A. Guerrero-Osuna, Gerardo Ornelas-Vargas, Rocío Carrasco-Navarro, Luis F. Luque-Vega, Emmanuel Lopez-Neri

**Affiliations:** 1 Unidad Académica de Ingeniería Eléctrica, Universidad Autónoma de Zacatecas, Zacatecas 98000, Mexico; 31126593@uaz.edu.mx (F.G.-V.); ornelas@uaz.edu.mx (G.O.-V.); 2 Department of Mathematics and Physic, ITESO AC, San Pedro Tlaquepaque, Jalisco 45604, Mexico; rociocarrasco@iteso.mx; 3 Centro de Investigación, Innovación y Desarrollo Tecnológico CIIDETEC-UVM, Universidad del Valle de México, Jalisco 45601, Mexico; luis.luque@uvmnet.edu (L.F.L.-V.); elopezneri@uvmnet.edu (E.L.-N.)

**Keywords:** smart home, internet of things, IoT device

## Abstract

As the development of systems in smart homes is increasing, it is of ever-increasing importance to have data, which artificial intelligence methods and techniques can apply to recognize activities and patterns or to detect anomalies, with the aim of reducing energy consumption in the main home domestic services, and to offer users an alternative in the management of these resources. This paper describes the design and implementation of a platform based on the internet of things and a cloud environment that allows the user to remotely control and monitor Wi-Fi wireless e-switch in a home through a mobile application. This platform is intended to represent the first step in transforming a home into a smart home, and it allows the collection and storage of the e-switch information, which can be used for further processing and analysis.

## 1. Introduction

Currently, the interest in Smart City (SC) and Smart Home (SH) are growing due to smartphone and internet use, alongside the rapid development of the Internet of things (IoT). Data-driven decision-making is helping to improve the efficiency of homes and cities, enabling a more sustainable mode of living that discourages wastefulness. This kind of change can arrive from top to bottom or from bottom to top. Therefore, it is crucial to involve the different major actors from the quintuple helix model: academia, industry, government, society and the natural environment.

In particular, the active participation of citizens is considered crucial to this strategy, and it is made clear that citizen participation and engagement form part of home smart solutions. A home is a place where people are in control and can feel safe. It can be seen as a place with security and control, a site of activity, and special for relationships and continuity. Besides, an SH is one in which a communications network links sensors, appliances, controls and other devices to allow for remote monitoring and control by occupants and others, in order to provide frequent and regular services to occupants and the electricity system; linking to physical and operational factors and assuming functionality beyond the usual boundaries of the home [1].

The important technological advances that have been presented in SH are related to the development of industry 4.0, which symbolizes the beginning of the fourth industrial revolution that represents the current trend of automation technologies in the industry. Mainly includes enabling technologies such as Cyber-Physical Systems (CPS), IoT, and Cloud Computing (CC), integrating the virtual space with the physical world [2]. Previously to the development of these technologies, installing and configuring an SH system was complicated because wired systems and infrared sensors were used, which were considered a real nuisance and affected user comfort. Currently, wireless technologies for SH, such as Wi-Fi, Bluetooth Low Energy (BLE) and ZigBee, help the development of systems in addition to allowing remote control through smartphone applications [3,4]. The classic SH, IoT, and cloud computing are the building blocks to achieve an advanced domotic integrated system. Each component brings its core attributes and technologies. IoT provides Internet connection and remote management of mobile devices through various sensors and actuators that measure the home conditions. In this way, they monitored the function of household appliances. Cloud computing provides computing power and storage space to develop, maintain, and run home services anywhere, anytime [5,6].

It is possible to see an SH as an automation process for different devices within a building, including monitoring control and automation for heating, ventilation, air conditioning (HVAC), lighting, electronics, household appliances, and security systems. This automation consists of the ability to schedule events for these devices either locally or remotely [7]. An SH system makes life at home more comfortable, but it also aims to reduce energy consumption. This is key for engaging actors interested in transforming homes into an SH, since electric energy consumption in the residential sector represents a quarter of the world’s total final energy consumption. It is essential in daily activities; therefore, practices and solutions that promote the saving and efficient use of energy must be included to correctly manage the energy supply in homes, preserving the natural environment [8].

SH systems could be integrated by a wide range of devices, such as smart switches and plugs, temperature, humidity and presence sensors, security cameras among others. Smart switches are devices that can be installed in an electrical appliance or house lights. They can be controlled and monitored with a mobile application from anywhere as long as the user has an active internet connection. In case of network failures, the switch can be manually controlled without using the internet, not affecting the user’s comfort. These devices are an alternative to reduce the electrical consumption of home lighting instead of changing the lights, or the user can even use both options to increase efficiency. Any attempt to reduce energy consumption consists of making an initial financial investment, recovered over time. However, it is essential to offer the user more alternatives to choose the best option.

An SH collects data from different devices used in everyday home activities like cooking, refrigeration, room illumination, heating water. The energy consumption collecting data is a critical process to improve efficiency practices in household appliances usage. According to the National Institute of Statistic and Geography (INEGI, by its initials in Spanish) statistics [9], the energy consumption of the house lighting is determined by the type of light installed. This statistic distinguishes between Compact Fluorescent Lamp (CFL), incandescent, and Light Emitting Diode (LED) bulbs. Their distribution at a national level is 162.2, 37.2, and 27.0 millions of bulbs, respectively. Moreover, the average time of use of light bulbs per day by home areas are (ordered from highest to lowest): garage 257 min, yard 230 min, kitchen 175 min, dining-living (DL) room 169 min, bedroom 148 min, bathroom 92 min. Table 1 shows a comparison of the produced lumens by different light bulb technologies and their power consumption [10]. Lumens are defined as the measurement of the total light emitted by a given source; this measuring unit is used to compare the brightness of light sources without being affected by the technology used, whether incandescent, fluorescent or LED.

An optimized light-filled space has to consider: (1) the Minimum Luminous level required by the activity carried out in the space (measured in lux units), and (2) the size area of the space. The electricity consumption in the space will rely on the chosen light bulb technology. Table 2 shows the total power consumption of an inhabited home using Incandescent, CFL and LED bulb technology.

It can be noted that LED technology power consumption is the lowest while the old incandescent bulbs and CFL consume approximately 2X and 7X, respectively. Therefore, LED bulbs reduce energy consumption thanks to their ability to provide more lumens with less power. National Survey on Energy Consumption in Private Homes (ENCEVI, by its initials in Spanish) survey shows that CFL, incandescent, and LED bulbs represent 72%, 16, and 12% of the total bulbs in Mexico, respectively [11]. Then, it is vital to face the challenge of changing the mindset of the Mexican citizen to focus on the importance of transforming their home into a smart home and how this will benefit its economy and the environment.

Therefore, introducing a new SH platform that increases user comfort by controlling and monitoring household appliances at home from anywhere and anytime as long as an active internet connection becomes the cornerstone when transforming a home into a smart home. Offering users adequate management of the lighting system in a home represents the first step towards an SH.

The contributions of this paper can be summarized as follows:

- Design and implementation of an e-Switch, based on a non-reactive approach to implement the SH concept, to optimize the use of installed household lighting networks. Controlling and monitoring each of the lights in the home locally or remotely in a more consciously and rationally way. This approach offers the user security and confidence when leaving home.
- Integration of IoT and a cloud environment to automate, control and monitor smart switches. Moreover, the data obtained by the platform are collected and stored for later analysis. Then, the platform is suitable and ready to offer information that can be used in artificial intelligence models for several purposes, such as reducing electrical energy consumption through the detection of patterns, searching for anomalies in the habits of the residents, among others.

The paper is organized as follows: Section 2 presents the existing works in SH and their main contributions. Section 3 describes the proposed platform presenting the model and implementation details. Section 4 shows the results obtained. In Section 5 the paper ends with the conclusions.

## 2. Related Work

The main application of the SH approach is to control and monitor household (smart) appliances and home features (lighting, windows, air-conditioning) to improve the domestic environment and optimize energy consumption. The households that apply the SH approach to optimize energy consumption have achieved to reduce about 5% energy consumption, promoting energy conservation and sustainable living [12]. The reduction rate was increased when more appliances were connected to smart devices, and their energy consumption was monitored.

Some standard SH applications follow a reactive approach design, using wireless sensor nodes distributed around the household to turn appliances on and off. These sensors send messages to the user if there is some source of energy that has surpassed some threshold (gas level, energy consumed) [6,13]. They report energy consumption, classifying it by type source or home appliance use [14,15]. They used to prevent damages to the household or their occupants [16,17], increasing levels of comfort of the occupants/users [18], and currently converting all the household appliances into reactive elements the smart home [19].

On the other hand, there are proposals where the control follows a non-reactive design, its main application is to predict solutions taking into account the inhabitants behavior to make the environment cozy and comfortable [20] and based on the user interaction with the system, so can it react when an intrusion to the home has occurred [21] or learning from the specific energy source load shifting characteristics as can be home renewable energy (wind turbines, solar panels, batteries) [22,23] or automatically deal with the random demand of consumers [24].

One of the main design decisions for non reactive designs, is the strategy to store data collected, so it can be accessible on real time, and taking in account time access to the established repository. Today is common the use of Android application or the Google assistant, using the Google Firebase cloud environment, which works as a remote database server to store the data collected from humidity and temperature sensors, which are used to read the house environment variables and keep it monitored [25] or using Emon-CMS as remote database server [26]. This approach enables user interaction and the development of software applications using artificial intelligence algorithms.

However, one of the main difficulties faced by researchers is to define the adequate amount of data for model validation and training purposes [27], since according the type of artificial intelligence technique used could require more data, as the neural network models [28,29] or requires multiple characterized information, as the activity recognition or natural language processing [30,31,32,33].

## 3. Design and Implementation of the E-Switch

Based on the articles in the related works section, we can see that most of the research involves platforms with integration of the IoT and the cloud. This section presents a system designed to monitor, control, and automate smart switches in a home environment. These switches can be installed on any appliance that is connected to the electrical grid of a house or building. The data generated by these devices are collected and stored in a database for later analysis.

### 3.1. Platform Scope and Objectives

We design and implement our platform following the guidelines and requirements that define an AAL system as defined by Zinner et al. in [34] and D. Popa et al. in [30], such as dedicated SLAs, upfront costs, usability, security, and interoperability. After analyzing these requirements, we mainly focused on parameters that we consider most important for implementing our platform: robustness, interoperability, security, and costs.

#### 3.1.1. Robustness

Robustness refers to the ability of the system to execute several processes simultaneously without generating errors. The platform’s core engine is based on the Firebase cloud that works as a remote server while recording and storing data. On the other hand, the engine on premises is an IoT component which is a device that has the function of a smart switch. The platform does not lose information in case of internet connection problems because the data are queued to be submitted when the connection is stable. Regarding the connection of devices, any change in the configuration of the switches can be supported without system failures and reconfigure the network mesh while staying online without losing its functionality [30].

#### 3.1.2. Interoperability

In an environment where numerous heterogeneous devices of different types and technical profiles operate, developing the ability to communicate with each other and make them easily accessible is one of the main challenges in IoT. IoT can be considered a highly dynamic and distributed networked system. From a system-level perspective, interoperability in the IoT ecosystem arises at each layer of the protocol stack due to the heterogeneity of devices, networks, and applications [35]. There are different communication technologies such as Wi-Fi, Bluetooth, ZigBee, or Z-Wave, aiming to provide solutions to a set of specific requirements for different application scenarios. Therefore, there is no and may never be a technology that meets all application scenarios [36]. Different devices and applications operate on their platforms without good compatibility with different providers in today’s IoT ecosystems. For example, a smart bracelet cannot interact with a smart bulb without the relevant private application provided by the same provider [37]. On this platform, the communication between the user and the devices is through a cloud service, the provider is in charge of managing the interoperability, and the devices do not necessarily have to be compatible in the physical, transport, and application layers, which allows having devices that operate with different technologies.

#### 3.1.3. Security

While SH platforms have led to significant technological advancements, the trust and acceptance of systems depend on the right balance between promoting innovation and ensuring user security and privacy. The large number of devices involved makes designing a secure system difficult because of the many potential attack points. Consequently, any solution must be able to handle data from a large number of devices without causing any loss of them, guarantee adequate security measures for transmitted data and prevent unauthorized external access. Security is an essential aspect of our platform. Firebase is certified according to the leading security and privacy standards. Some services like Firebase Authentication use the data to enable end-user authentication and make managing user account easier. It also uses user-agent strings and IP addresses to provide additional security and prevent abuse during registration and authentication. Regarding privacy, our platform only works with a data controller of personal information about users (Emails, passwords), and Firebase acts as the data processor; this means that the information is under our control and we have the responsibility about it [38].

Currently, the IoT platform is using the security layer provided by Firebase, which Google Cloud backs. However, to provide a more solid security layer is interesting to consider the work done in [39]. Eventually, this can trigger different applications with a high level of security and reliability for Smart Homes.

#### 3.1.4. Costs

When we talk about the benefits of implementing an SH in terms of costs, we must consider the user’s investment cost and what exactly they can get with our platform. Residents have the opportunity to monitor and automate some domestic services, either locally or remotely. This ensures proper handling of the devices to which the smart switch connects. It could be a water pump, a household appliance, or the lighting of the building. With these devices connected to the platform, users can reduce costs in electricity, water, and fossil fuels.

### 3.2. Platform Architecture

As mentioned above, the cloud environment takes care of the interoperability of IoT devices; this facilitates the connection between devices that use different wireless technologies. However, integrating multiple communication protocols on a single platform would result in a complex control system. Therefore, we propose to use devices that only handle a specific physical layer. We analyzed different existing technologies for SH, such as ZigBee, BLE, Wi-Fi. We decided to use Wi-Fi, because according to INEGI [40], 70.1% of the population aged six years or more in Mexico is an internet user, and 20.1 million households (56.4% of the national total) have an internet connection. This allows cloud access to be carried out through a Wi-Fi connection to a Router connected to the internet or mobile data through a smartphone, which can also function as Wi-Fi access points. This offers us a high availability for connecting the smart switches.

**Figure 1:**
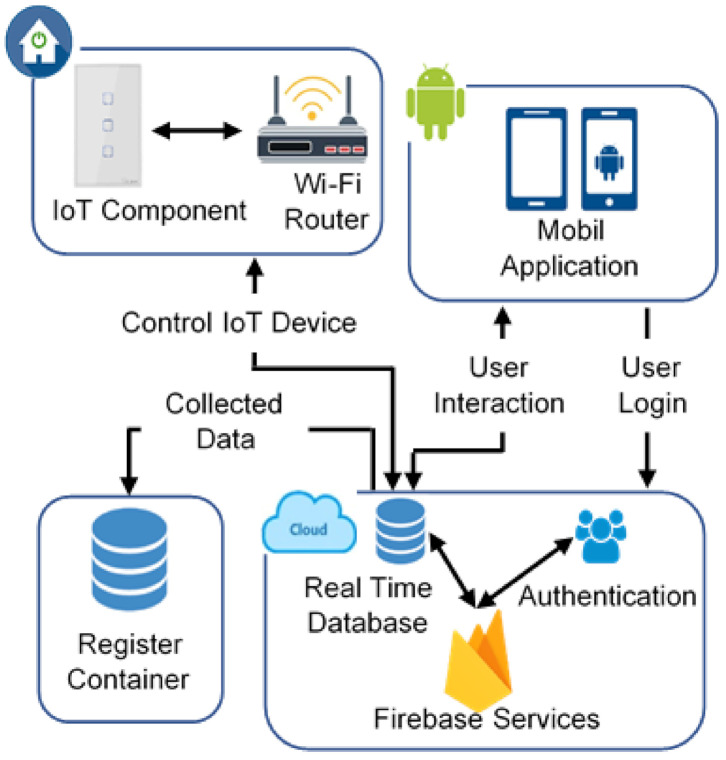
Platform architecture.

Figure 1 illustrates the proposed architecture, which is composed of four main components: an IoT component that has the function of a Wi-Fi smart switch, the Firebase cloud environment, a graphical user interface to interact with the system that is an Android application, and the registry container, which is responsible for collecting data from the platform. Each of these components can be implemented separately and works as an independent service. However, they can communicate between them. A smart switch is installed in the electrical grid of a building. It is connected using Wi-Fi wireless technology to a Router. The switch establishes a connection to the internet and with Firebase. The Android application is synchronized with Firebase; it provides a user interaction (Switch control and monitoring) and authentication to provide an administration in user accounts. The register container collects information from IoT devices like location (Kitchen, Living Room, Bedroom), switch status (ON/OFF), and date and time when the status change was made.

#### 3.2.1. Iot Component

Wi-Fi smart switches are devices capable of remotely connecting or disconnecting any electrical device connected to the power outlet in a building using the internet. In this case, the switch acts as an intermediary between the electrical energy and the appliance. By controlling wired devices through wireless devices, greater flexibility and extensibility are achieved. Since its operation is easy, it can be applied to any electrical appliance in the home, and specialized personnel is not needed for its operation and installation. Furthermore, the device can be controlled even without the use of the internet. If users do not have internet access, they can manually control household appliances as with any conventional electrical switch without affecting user comfort in case of network problems [41].

There are different devices for home automation in the market, which have the function of a smart switch. These options are based on their price, wireless technology, and the supplier of the product. Since we needed a device with Wi-Fi technology, we analyzed commercial products and selected the Sonoff product from the Itead company. They offer the possibility of modifying their devices to use our platform or system, which is essential to generate our information. Sonoff allows a wide range of connections with all types of household appliances, LED spotlights, fluorescent lamps, LED reflectors, fans, or air conditioning. A Sonoff device comprises four blocks: Power supply (3.3v), serial (Tx, Rx), relay, an ESP8266 chip that works as the controller, and wireless communication module for 2.4 GHz networks.

**Figure 2:**
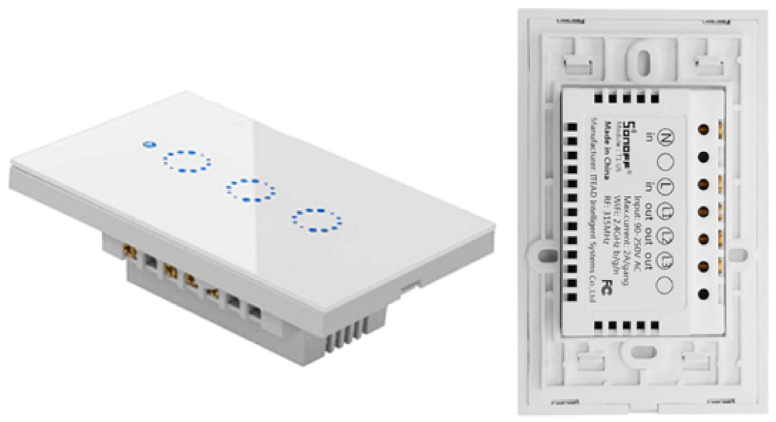
Sonoff T1 3-Gang.

**Figure 3:**
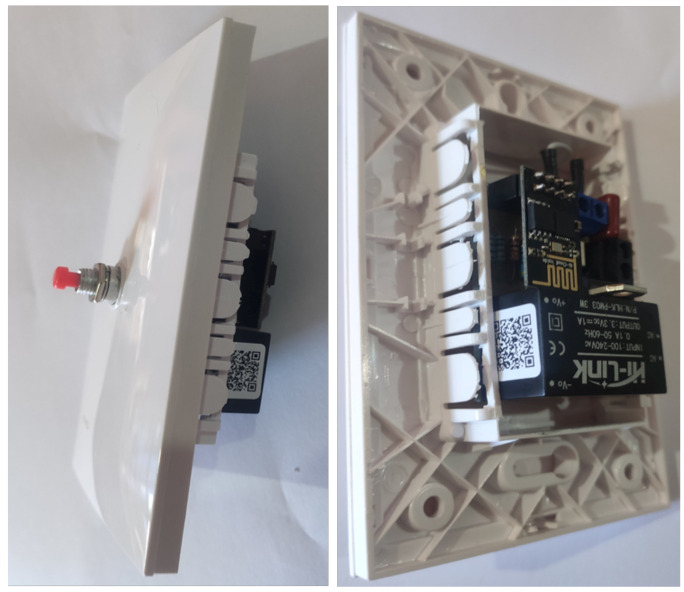
e-Switch.

After the analysis period of commercial devices on the market, we observed that most of them have relays to isolate the control from the electrical grid, so we decided to design our smart switch but using an optocoupler since it has several notable advantages compared to the electromechanical relays and contactors: they are faster, quieter, lighter, more reliable, do not wear out, are immune to shock and vibration, can switch high currents and high voltages without producing arcs or ionizing the surrounding air, and they generate very little interference. The optocoupler has a phototriac output stage and the respective components for coupling to the electrical network. Like with the sonoff, the ESP8266 chip was used, and a push-button is used to change the switch state. Figure 2 shows the sonoff T1 3 Gang device [42]. This switch has three outputs, which are known as channels, whereas the switch designed by us is shown in Figure 3, which only has one channel, and we call it e-Switch.

#### 3.2.2. Cloud Service

Firebase is a mobile platform created by Google, whose primary function is to develop and facilitate high-quality web applications and mobile applications. Firebase offers various services for application development. For specific purposes of our platform, we use the real-time database and authentication for user registration [43].

Firebase provides a NoSQL real-time database; it stores all data as JavaScript Object Notation (JSON). Unlike traditional databases, there are no tables or registers. All data added to Firebase is added to the JSON tree and becomes a separate node in the JSON structure, with an associated key. Keep in mind that how data are stored in the system is paramount to ensure efficient database queries and ensures that the system scales as demand grows. In addition, the service provides an SDK that allows information from the web or mobile applications to be synchronized and stored in the cloud.

On the other hand, the database is also accessible through a REST API that uses the SSE (Server-Sent Events) protocol, an API to create HTTP connections. Instead of typical HTTP requests, data synchronization is used (every time the data changes, connected devices receive that update within milliseconds) [44]. Real-time synchronization allows users to monitor the IoT component from the mobile application in real-time. Each time the user modifies it, the information is stored in the cloud, and the IoT component is notified simultaneously. An exciting feature of this database is that if a user makes changes and loses their Internet connection at the same time, the platform’s SDK uses a local cache on the device where it saves these changes. Once the user is back online, the local data are automatically synced.

For registration, we enable the Firebase Authentication service to authenticate users using only client-side code. It includes authentication through login providers such as Facebook, GitHub, Twitter, Google, Yahoo, Microsoft, and the classic login methods using email and password [45]. When the user accesses the Android application for the first time, a user account must be created with the corresponding credentials (email and password). This new account is stored as part of the Firebase project and can be used to identify a user in the application. In this way, the user’s information can be managed in Firebase. If the user changes their phone or uninstalls the application, their information is still available as it is stored in the database.

#### 3.2.3. Mobile Application

**Figure 4:**
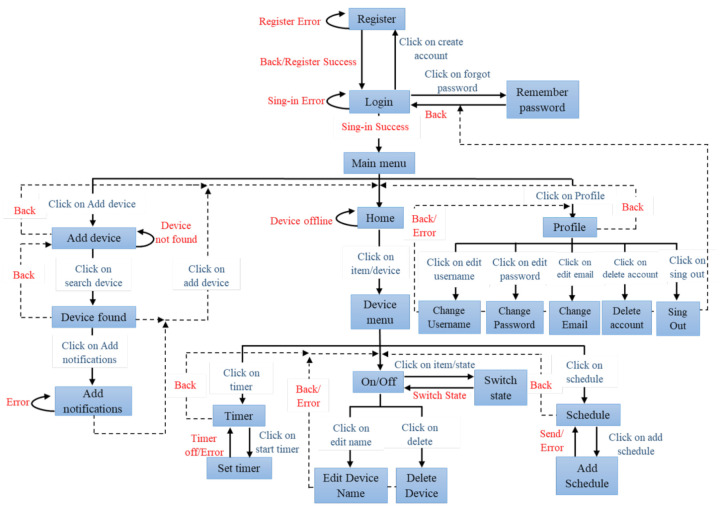
Mobile app window diagram.

Firebase is available for different platforms such as iOS, Android, and Web. In this first stage of our platform, we include an application with an Android system. The application shows the status and configuration of the current environment, allows the user to control and monitor the IoT components remotely. The first time a user accesses the application, it is necessary to authenticate with an email from any domain. In this way, they can register and create a new account. Once the user has logged in, they can navigate through different menus and sections, which can be seen in Figure 4.

**Figure 5:**
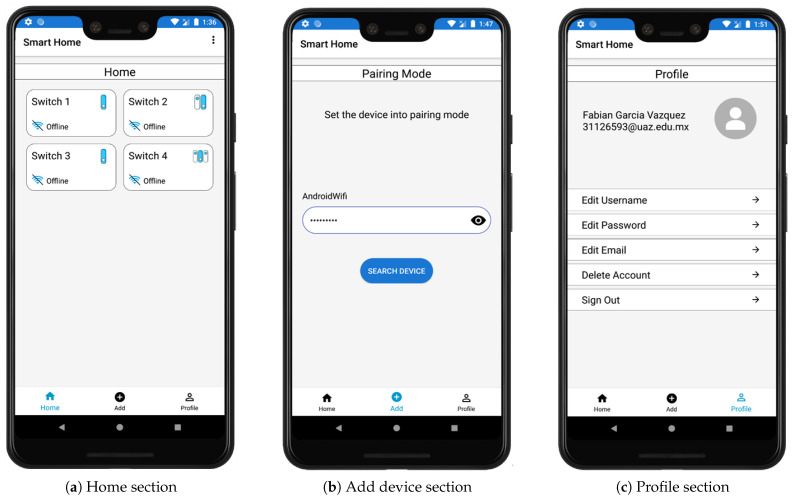
Android app main menu.

The main menu of the application has the following sections:

- Home—This section shows the list of switches that the user has added so far. In the first instance, the user will not have any device, so it is necessary to add it. The list shows the devices with a specific name which is selected by the user, as well as their connection status (Connected or Offline), see Figure 5a.
- Add—Add a device to the Home section. The switch must be placed in pairing mode according to the user manual and enter the network’s password of the network to which the user wants to connect the device. Once the device is found, an identifying name is placed on it and displayed in the Home section, see Figure 5b.
- Profile—Account administration, change username, password, email, delete account and sign out, see Figure 5c.

**Figure 6:**
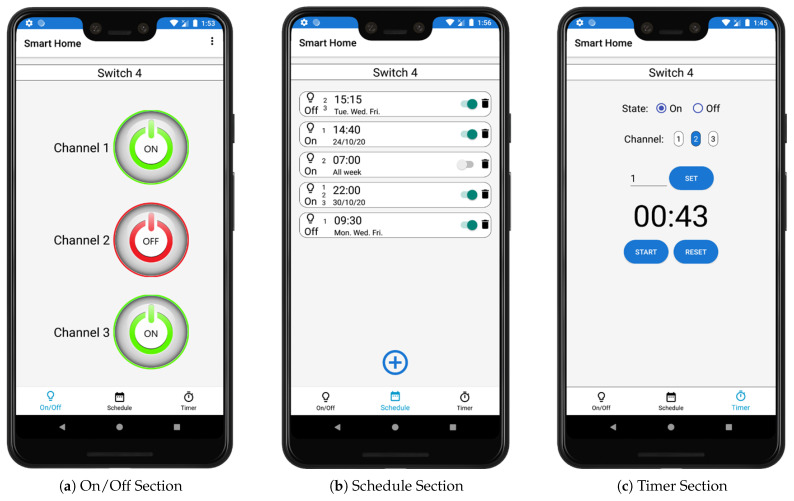
Android app device menu.

Maintaining the system’s interoperability, we designed the application to support switches with different outputs or channels. The device’s code must include its number of channels. When the pairing is carried out, the application detects the available channels and adds them to the control and monitoring interface automatically. This interface is accessed through the list in the Home section, which presents the following sections:

- On/Off—It allows control and monitoring of the device. If the switch is turned on or off manually from the premises, it is reflected in this section. In addition, in this section, we add options to increase user comfort, allowing them to edit the name or delete the device, see Figure 6a.
- Schedule—In this section, it is possible to establish synchronization times for turning the switches on and off at a specific date and time, see Figure 6b.
- Timer—In this section, the switch can be turned on or off automatically for a specified time, see Figure 6c.

**Figure 7:**
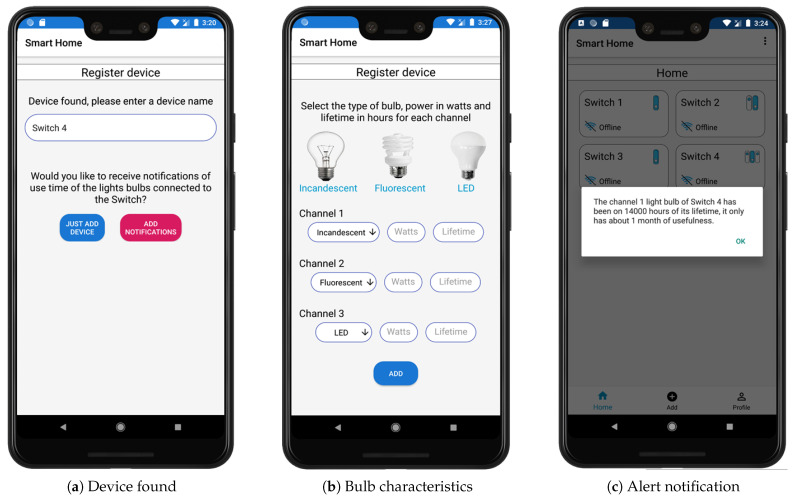
Add device to Android app.

Considering that the device is found during pairing, an identification name must be added to the switch. The user is offered an option to receive notifications of use time of the bulbs connected to the Sonoff or e-Switch, as can be seen in Figure 7a. If the user does not want to add notifications to the switch, the device is only added to the home section. If notifications are required, the app displays a panel to add characteristics of the bulbs depending on the number of channels, such as the type of technology (incandescent, fluorescent, or LED), the power, and the lifetime of the bulbs, as can be seen in Figure 7b. This information is specified on the packaging of each bulb, although in the case of incandescent bulbs, it is more difficult to find such information. The notification consists of an alert that indicates when the bulb connected to a switch is close to ending its useful life. This notification is based on the use of the bulb. The platform establishes an approximate remaining lifetime, as can be seen in Figure 7c.

#### 3.2.4. Register Container

Firebase’s real-time database works as a remote server for IoT components and applications; however, data stored in Firebase is constantly changing due to switch control; therefore, there is no record and compilation of that information. For this reason, we decided to add another component to the platform called a register container, which has the function of registering all the platform’s data in a local database. We implement this container in the Python language, since similar to the Android application.

Firebase provides the corresponding SDK to access the real-time database from the Python environment. In this first stage of the platform, there is not yet a sufficient amount of data, so it is not possible to apply artificial intelligence algorithms, but the information recorded is prepared and directed towards this approach. In the register container, data from the switches are related to the location, channel, switch status, and the date and time the status change was made.

The information records can be made in different ways; one of them is to establish a sampling period to obtain the data, but in this case, it is useless because we would be reading the database from time to time, and the switches states may remain at the same value. As a result, it is more convenient to record the data only when there is a state change, whereas the sampling period can be added in information pre-processing.

## 4. Results

**Figure 8:**
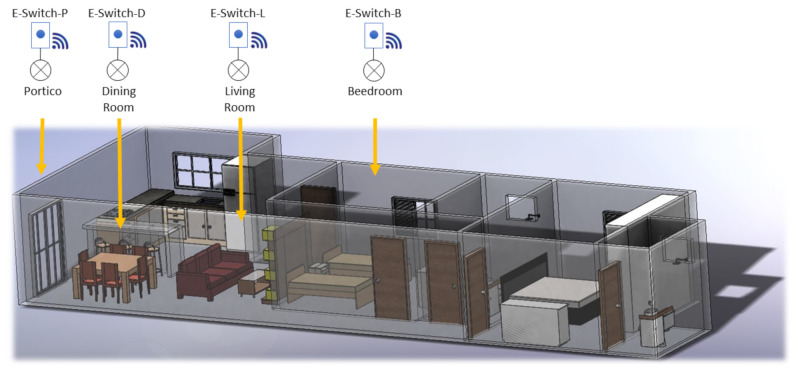
e-Switches home distribution.

In order to evaluate the performance of the complete solution, four e-Switches were installed to control and collect lightning data from an apartment using 10 W LED bulbs. The specific home’s areas selected are: portico, bedroom, living room, and dining room lights in which the e-Switch-P, e-Switch-B, e-Switch-L, and e-Switch-D were installed, respectively (see Figure 8). Data from these IoT devices is collected from 6th September to 6 December 2020. Table 3 shows the obtained information stored in the register container gathered by these devices in sta period of 92 days.

On the other hand, the energy use of any electrical device can be estimated by multiplying its power rating (PR) by its daily duration time of use (DU):

(1) E(Wh)=PR(W)·DU(h)

Then, the total appliance daily energy consumption $T_{DEC}$ considering *m*—electrical devices and *n*—days can be defined as:

(2) $$T_{DEC}=\sum_{i=1}^{m}\sum_{j=1}^{n}E_{ij}.$$

Table 4 shows the average time turn on and the average times the light bulb turns on per day, in addition to the average power consumption per day and total generated by the bulbs in the areas where the switches were installed.

**Figure 9:**
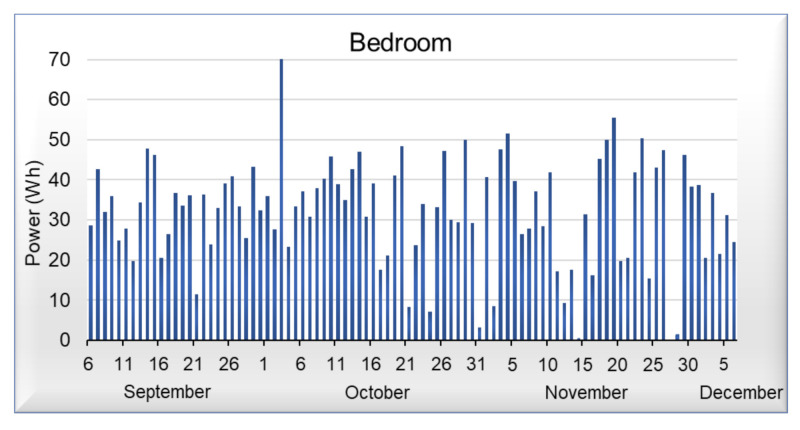
Power consumed by the bedroom light.

**Figure 10:**
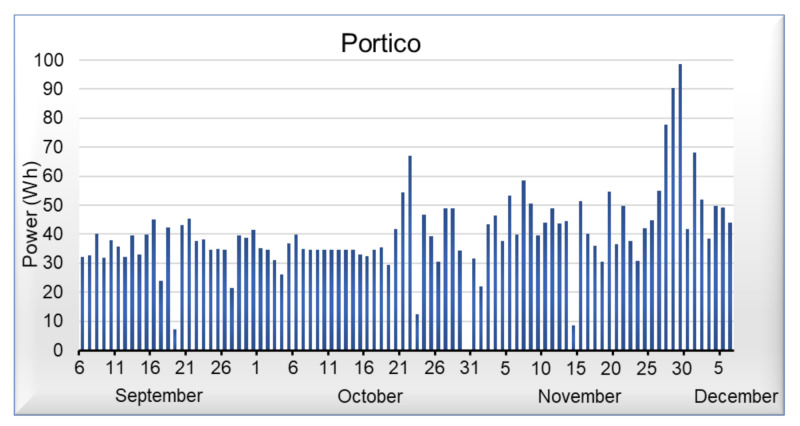
Power consumed by the portico light.

**Figure 11:**
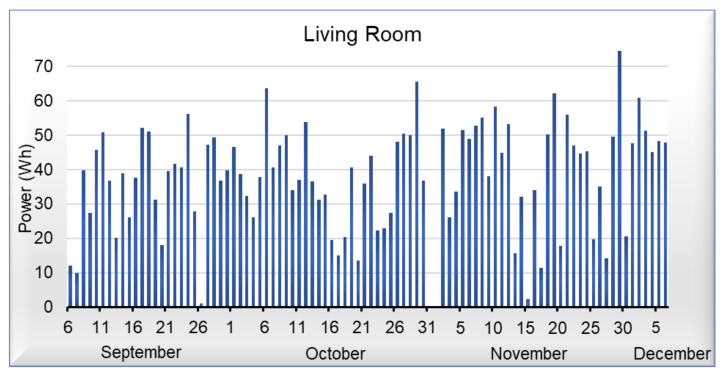
Power consumed by the living room light.

**Figure 12:**
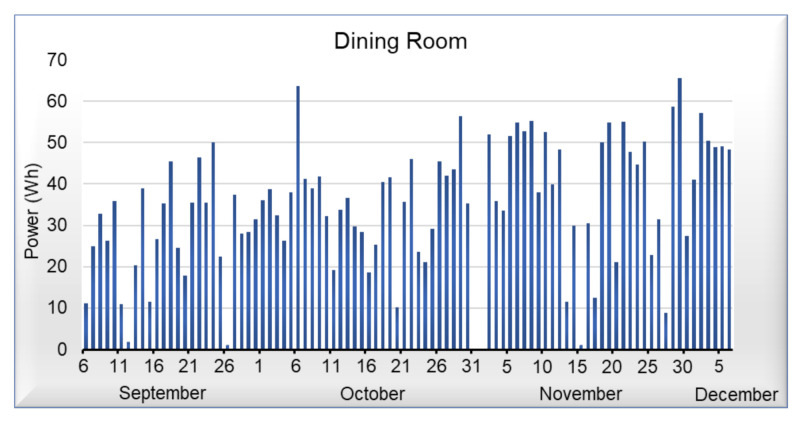
Power consumed by the dining room light.

Figure 9, Figure 10, Figure 11 and Figure 12 shows data corresponding to the energy consumed per day. Although we have not yet added the artificial intelligence algorithms to the platform, we can observe that in the graphs, there are trends and behaviors that can be considered anomalies or pattern recognition, such as the case of Figure 9, where energy consumed by the light of the room, commonly oscillates between 20 and 45 Wh per day, but on October 5 it shows an anomaly, which represents an increase in energy demand. On the other hand, in Figure 10 that corresponds to the portico, we can see that the energy demand has an average of 35 Wh per day; however, it is not possible to detect an anomaly or pattern with a visual analysis in this area. Regarding the living room and dining room of Figure 11 and Figure 12, we can observe that they have similar behavior; in these areas, we can detect an average of 40 Wh during the week, whereas on the weekends, there is a decrease in energy.

## 5. Conclusions

Currently, SH is composed of various sensors and actuators from different manufacturers, which makes the connection between them difficult as they operate with different communication technologies and protocols. Cloud environments take care of system interoperability, allowing interaction between IoT devices from different providers, which increases the system’s robustness without affecting its security. This system aims to increase the resident’s comfort by controlling and monitoring the main electrical appliances in the home, which represents adequate management in services such as electricity, water and fossil fuels. This hardware is well developed but lacks tools to transform home automation devices into smart ones. Artificial intelligence must be integrated to develop fully autonomous devices and make decisions based on the habits of residents.

In this paper, we describe the implementation of a platform for the remote control and monitoring of Wi-Fi smart switches within a home, offering an alternative for the optimal management of electrical energy. We installed the switches in an apartment’s lighting areas. We obtained data corresponding to the switching of the lights in those areas. These data are stored and collected in a register container, reflecting people’s habits regarding the frequency with which they turn on or off the lights. This platform allows us to generate our database. We will seek to perform an analysis that allows the application of artificial intelligence techniques and methods to recognize and detect anomalies. In this way, we can add intelligence to the devices so that they act completely autonomously.

## Figures and Tables

**Table 1:** Comparison of the produced lumens and the power consumption from different light bulb technologies.

Lumen (lm)	Incandescent (W)	CFL (W)	LED (W)
250	25	4–9	3
450	40	9–13	4–5
800	60	13–15	6–8
1100	75	18–25	9–13
1600	100	23–30	16–20
2000	125	30–40	20–25
2600	150	30–55	25–28

**Table 2:** Power consumption comparison of bulb technologies in an inhabited home.

	Lux (lx)	Area (m${}^{2}$)	Lumen (lm)	Incandescent (W)	CFL (W)	LED (W)
Bedroom	150	9	1350	100	25	15
Bathroom	100	3.5	350	40	9	5
Portico	100	2	200	25	5	3
Kitchen	200	8	1600	100	25	15
DL room	300	9	2700	150	42	23
			Total	415	106	61

**Table 3:** Platform register container.

Area	Switch	Channel	State	Date	Time
Bedroom	e-Switch-B	Channel1	TRUE	9 June 2020	20:06:56
Bedroom	e-Switch-B	Channel1	FALSE	9 June 2020	23:00:11
Portico	s-Switch-P	Channel1	TRUE	9 June 2020	20:15:02
Portico	e-Switch-P	Channel1	FALSE	6 September 2020	23:31:02
Living Room	e-Switch-L	Channel2	TRUE	9 June 2020	20:16:49
Living Room	e-Switch-L	Channel2	FALSE	9 June 2020	21:11:46
Living Room	e-Switch-L	Channel2	TRUE	9 June 2020	21:51:16
Living Room	e-Switch-L	Channel2	FALSE	9 June 2020	21:57:12
Living Room	e-Switch-L	Channel2	TRUE	9 June 2020	22:30:22
Living Room	e-Switch-L	Channel2	FALSE	9 June 2020	22:31:03
Living Room	e-Switch-L	Channel2	TRUE	9 June 2020	22:45:02
Living Room	e-Switch-L	Channel2	FALSE	9 June 2020	22:56:53
Dining Room	e-Switch-D	Channel3	TRUE	9 June 2020	20:16:50
Dining Room	e-Switch-D	Channel3	FALSE	9 June 2020	21:11:47
Dining Room	e-Switch-D	Channel3	TRUE	9 June 2020	22:30:22
Dining Room	e-Switch-D	Channel3	FALSE	9 June 2020	22:31:03
Dining Room	e-Switch-D	Channel3	TRUE	9 June 2020	22:45:02
Dining Room	e-Switch-D	Channel3	FALSE	9 June 2020	22:56:54
⋮	⋮	⋮	⋮	⋮	⋮
Bedroom	e-Switch-B	Channel1	TRUE	12 June 2020	9:20:18
Bedroom	e-Switch-B	Channel1	FALSE	12 June 2020	22:41:09
Bedroom	e-Switch-B	Channel1	TRUE	12 June 2020	23:24:56
Bedroom	e-Switch-B	Channel1	FALSE	12 June 2020	23:29:42
Portico	e-Switch-P	Channel1	TRUE	12 June 2020	18:59:31
Portico	e-Switch-P	Channel1	FALSE	12 June 2020	23:24:19
Living Room	e-Switch-L	Channel2	TRUE	12 June 2020	18:20:11
Living Room	e-Switch-L	Channel2	FALSE	12 June 2020	23:08:45
Dining Room	e-Switch-D	Channel3	TRUE	12 June 2020	18:19:14
Dining Room	e-Switch-D	Channel3	FALSE	12 June 2020	23:10:41

**Table 4:** Power consumption calculated based on register container.

Area	Average Time Turn on Per Day (h)	Average Times the Bulb Turns on Per Day	Average Power Consumption Per Day (Wh)	Total Power Consumption (Wh)
Bedroom	3.22	3	32.17	2959.70
Portico	4.03	2	40.33	3710.43
Living Room	3.77	2	37.69	3467.04
Dining Room	3.46	2	34.59	3182.58

h = hours, Wh = Watt-hour.

## Data Availability

The data presented in this study are available in Table 3.
